# Quantitative parameters in dynamic contrast-enhanced magnetic resonance imaging for the detection and characterization of prostate cancer

**DOI:** 10.18632/oncotarget.24652

**Published:** 2018-03-23

**Authors:** Cheng Wei, Bowen Jin, Magdalena Szewczyk-Bieda, Stephen Gandy, Stephen Lang, Yilong Zhang, Zhihong Huang, Ghulam Nabi

**Affiliations:** ^1^ Division of Cancer Research, School of Medicine, University of Dundee, Ninewells Hospital, Dundee DD1 9SY, UK; ^2^ Department of Clinical Radiology, Ninewells Hospital, Dundee DD1 9SY, UK; ^3^ Department of Medical Physics, Ninewells Hospital, Dundee DD1 9SY, UK; ^4^ Department of Pathology, Ninewells Hospital, Dundee DD1 9SY, UK; ^5^ School of Science and Engineering, University of Dundee, Dundee DD1 4HN, UK

**Keywords:** prostate cancer, dynamic contrast-enhanced magnetic resonance imaging, multi-parametric magnetic resonance imaging, kinetic models

## Abstract

**Objectives:**

to assess the diagnostic accuracy of quantitative parameters of DCE-MRI in multi-parametric MRI (mpMRI) in comparison to the histopathology (including Gleason grade) of prostate cancer.

**Patients and methods:**

150 men with suspected prostate cancer (abnormal digital rectum examination and or elevated prostate-specific antigen) received pre-biopsy 3T mpMRI and were recruited into peer-reviewed, protocol-based prospective study. The DCE-MRI quantitative parameters (*K^trans^* (influx transfer constant) and *k_ep_* (efflux rate constant)) of the cancerous and normal areas were recorded using four different kinetic models employing Olea Sphere (Olea Medical, La Ciotat, France). The correlation between these parameters and the histopathology of the lesions (biopsy and in a sub-cohort 41 radical prostatectomy specimen) was assessed.

**Results:**

The quantitative parameters showed a significant difference between non-cancerous (benign) and cancerous lesions (Gleason score≥3+3) in the prostate gland. The cut-off values for prostate cancer differentiation were: *K^trans^* (0.205 min^−1^) and *k_ep_* (0.665 min^−1^) in the extended Tofts model (ET) and *K^trans^*(0.205 min^−1^ and *k_ep_* (0.63 min^−1^) in the Lawrence and Lee delay (LD) models respectively. The mean *K^trans^* value also showed a difference between low-grade cancer (Gleason score=3+3) and high-grade cancer (Gleason score ≥ 3+4). With the addition of DCE-MRI quantitative parameters, the sensitivity of the PIRAD scoring system was increased from 56.6% to 92.1% (*K^trans^*_ET), 93.1% (*k_ep_*_ET), 91.0%, (*K^trans^*_LD) and 89.4% (*k_ep_*_LD).

**Conclusion:**

Quantitative DCE-MRI parameters improved the diagnostic performance of conventional MRI in distinguishing normal and prostate cancers, including characterization of grade of cancers. The ET and LD models in post-image processing analysis provided better cut-off values for prostate cancer differentiation than the other quantitative DCE-MRI parameters.

## INTRODUCTION

Prostate cancer is the fifth leading cause of death from cancer in males (6.6% of total male deaths). An estimated 1.1 million men worldwide were diagnosed with prostate cancer (PCa) in 2012 (World Health Organization, GLOBOCAN 2012) [1]. Multi-parametric MRI (mpMRI) enables anatomical and molecular assessment of prostate cancers, including their cellularity and vascularity [2]. It has been suggested and widely used as a useful diagnostic modality for both localization and characterization of the PCa in many studies [2–9].

The mpMRI examination of the prostate typically consists of three stages, namely (i) anatomical imaging, (ii) tissue diffusion and (iii) tissue perfusion. The anatomy of the prostate and surrounding tissues can be visualized via the use of well-established qualitative MR techniques, such as T1-weighted (T1W) and T2-weighted (T2W) images. Suspicions within the prostate in T2WI are often seen as subtle areas of hypo-intense signal relative to the surrounding healthy tissues. These anatomical assessments are supplemented by diffusion-weighted imaging (DWI), which can probe the diffusion of extracellular and intracellular water molecules within the prostate tissues and highlight areas of low apparent diffusion coefficient (ADC), which may provide evidence for a diagnosis of prostate cancer [3]. In addition to DWI, gadolinium (Gd)-based techniques are also used to monitor the passage of a Gd contrast agent through the prostate tissues by dynamic contrast-enhanced MRI (DCE-MRI) [2]. The post-processing analysis of these images often studies semi-quantitative features associated with the contrast enhancement, such as the contrast up-slope, time-to-peak enhancement, and so forth [10, 11]. These assessments are included within the current radiological grading scheme for prostate cancer, which is the Prostate Imaging Reporting and Data System (PI-RADS) scoring system. The PI-RADS is grounded on the five-grade Likert-like scale, evaluating the risk of clinically significant prostate cancer [12–14]. Although the semi-quantitative measures alone do not necessarily form clear-cut correlations with pathophysiologic features [4]. By examining the contrast wash-in and wash-out more quantitatively and by using pharmacokinetic modelling techniques, it is believed that the resulting indices may better reflect the microvascular properties within the tissue [10, 11]. Most studies in this area are from patients where MR imaging has been obtained after biopsies of prostate (with confirmed diagnosis) or histopathology of radical prostatectomy specimens are not aligned to imaging. There is no data or reported study on biopsy naïve prostate imaging, specifically using prospective protocol based 3T MR imaging. Moreover, validation data in which MRI sectioning of prostate alignment with histopathological processing using 3-D mold fabrication has not been reported.

In this study, we had the following objectives:

- To prospectively assess the diagnostic accuracy of the quantitative parameters of DCE-MRI (mpMRI) in comparison to histopathology by independent observers in men with pre-biopsy imaging.
- To establish “cut-off” values for pharmacokinetic parameters that may distinguish benign and cancerous prostate tissue, using a new post-processing analysis software algorithm.

## RESULTS

**Figure 1:**
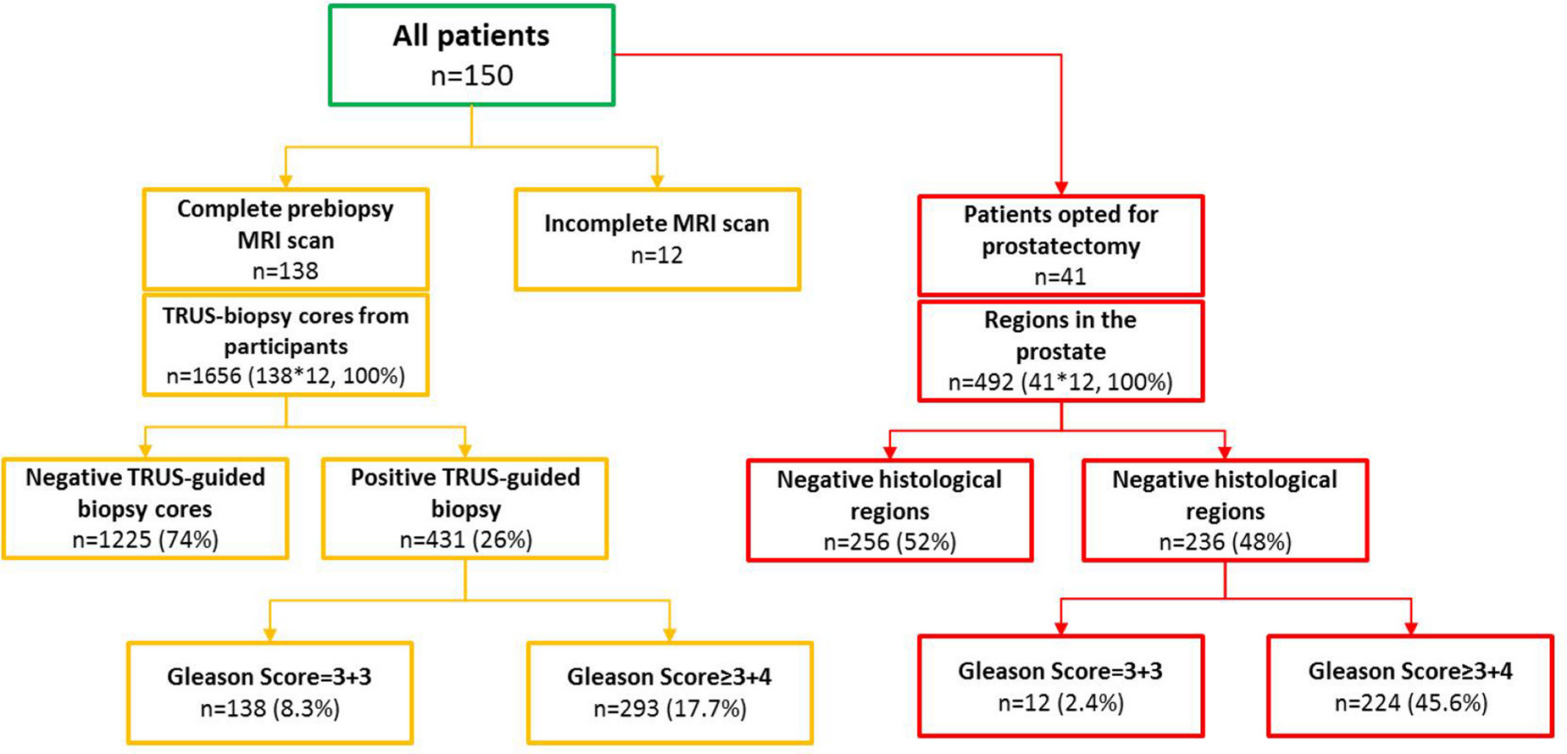
Flow of patients through the study

Of the 150 patients recruited, pre-biopsy mpMRI was successfully performed in 138 patients. 12 patients ended up with incomplete or non-diagnostic MRI scans. Of those patients who completed the study, 1656 biopsy regions (12 regions x 138 patients) were analyzed. Positive TRUS-guided biopsy was identified in 26% of cases (n=431 regions), Gleason Score 3+3 were found in 8.3% of all biopsy regions and Gleason score ≥ 3+4 were found in 17.7% of all biopsy regions. And the remaining 74% (n=1225 regions) were TRUS-guided biopsy negative. In addition, 41 patients received prostatectomy, corresponding of all 492 histological regions (12 regions x 41 patients) were analyzed. n=256 (52% of all histological regions) were deemed to be histologic negative. And n= 236 (48% of all histological regions) were deemed to be histologic positive, which contains Gleason score = 3+3 (n=12) and Gleason score ≥ 3+4 (n=224). A summary of these results can be found in Figure 1.

**Figure 2:**
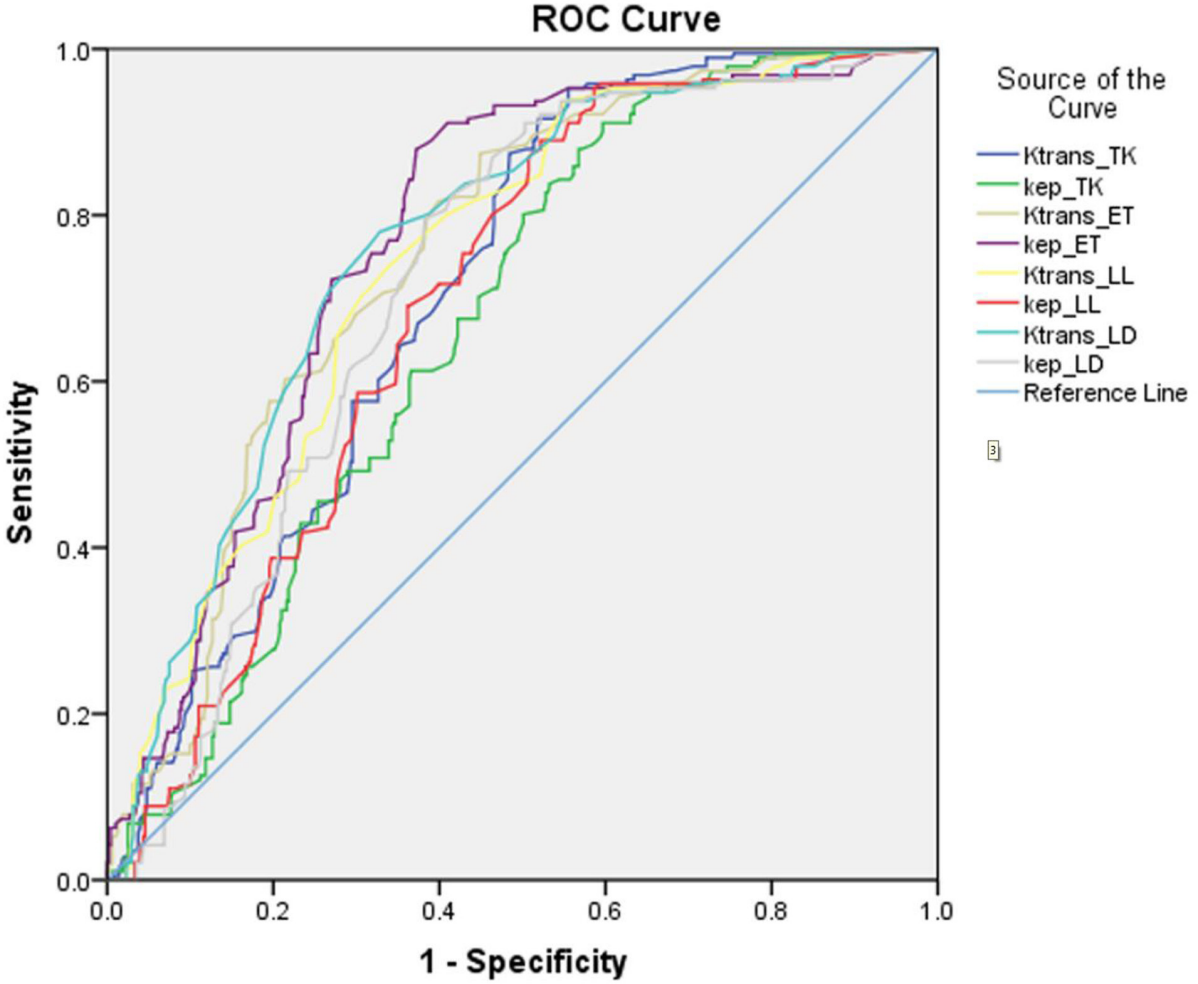
The ROC curves of Ktrans and kep in four different kinetics models for performance in benign prostate tissue and PCa (Gleason score≥3+3)

Biopsy histopathology results were initially used as the reference standard in the main cohort. The comparison between quantitative DCE parameters illustrates that *K^trans^* and *k_ep_* have better diagnostic performance for PCa than *v_e_* and *v_p_*. Amongst the four models, the Extended Tofts (ET) and Lawrence & Lee delayed (LD) model had a higher area under curve than the other two models (Figure 2, Table 1); this indicates these two models provide better diagnostic performance in differentiating cancer from benign prostate tissue. Compared to the other two models, the mean *K^trans^* and *k_ep_* values of the ET and LD models for the cancer and non-cancer groups also show a greater distinction. Thus, the *K^trans^* and *k_ep_* cut-off values of the ET and LD models were selected separately as a DCE parameter grading system. The cut-off values of both models show high similarity.

**Table 1:** Patients selection and baseline characteristics

Patients selection	
Inclusion criteria	Exclusion criteria
✓ Males between the age of 40-75	× Prior prostatic biopsy
✓ With at least 10 years’ life expectancy	× Contraindication to biopsy
✓ Referred from primary care:	× Poor general health and life expectancy < 10 years
∙ With clinically localised PCa: PSA>2.5 and <20	× Previous diagnosis of acute prostatitis × History of prostate cancer
∙ And/or abnormal DRE examination but <T3 disease	× Prior transurethral prostatectomy
	× Contraindication to MRI
	× Contraindication to MRI
**Patients characteristics**	
Patients number	**150**
Mean age (range)	62.5 (48-75)
Mean PSA level, ng/ml (range)	8.5 (2.7-20)
Mean prostate volume, cc (range)	58 (13-170)
Patients with incomplete DCE sequences (%)	12 (8%)
MRI negative patients (%)	30 (20%)
MRI positive patients (%)	108 (72%)

**Figure 3:**
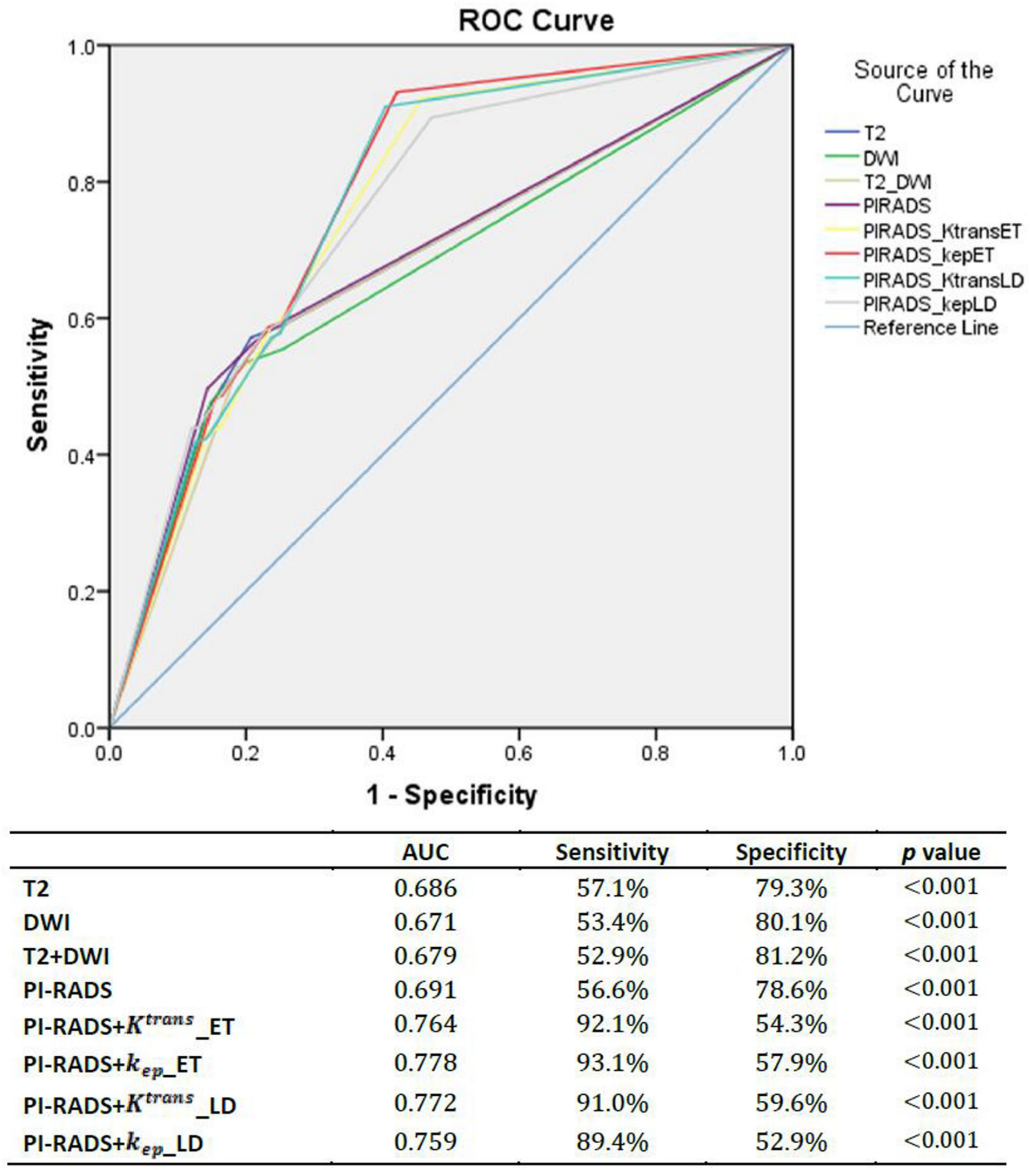
The ROC curves of T2, DWI and PIRAD scoring system and the accession of DCE-MRI scoring system which based on the cut-off value of different parameters for benign prostate tissue and PCa (based on biopsy results)

T2 and DWI scores were analyzed by experienced radiology consultants. The sensitivity and specificity of the T2 and DWI scores for differentiating cancer and benign prostate tissue were 57.1%, 79.3% and 53.4%, 80.1%, respectively. The combination of the T2 and DWI scores did not show any significant improvement. The sensitivity and specificity of the PI-RADS were similar to those of T2 and DWI. However, the addition of the DCE-MRI parameter cut-off value to PI-RADS significantly enhanced the sensitivity and specificity as the AUC value distinctly increased (Figure 3).

**Figure 4:**
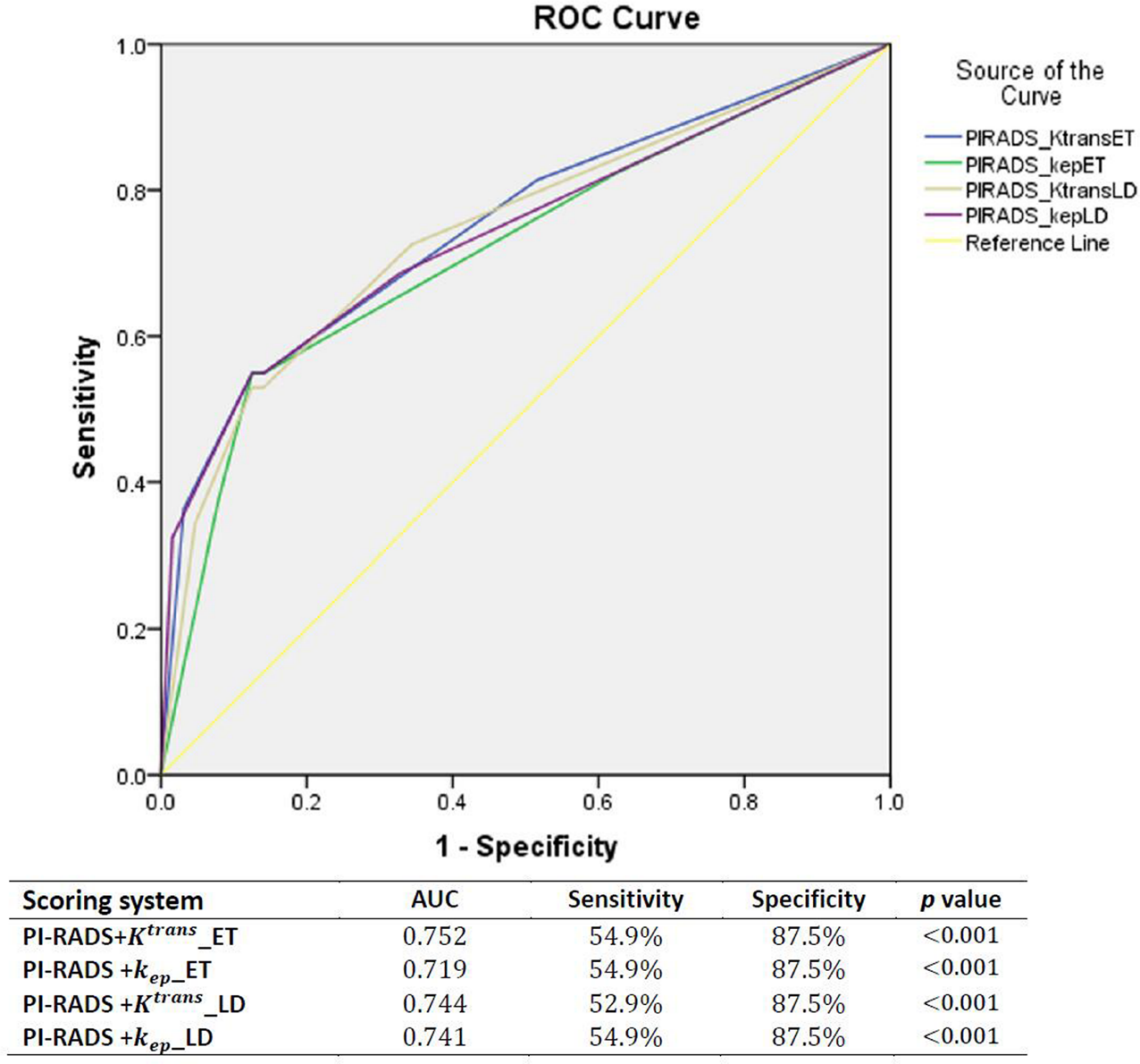
The ROC curves and AUCs of the scoring system (PIRADS+DCE parameters) which used the Gleason score from histology (radical prostatectomy as reference standard)

**Figure 5:**
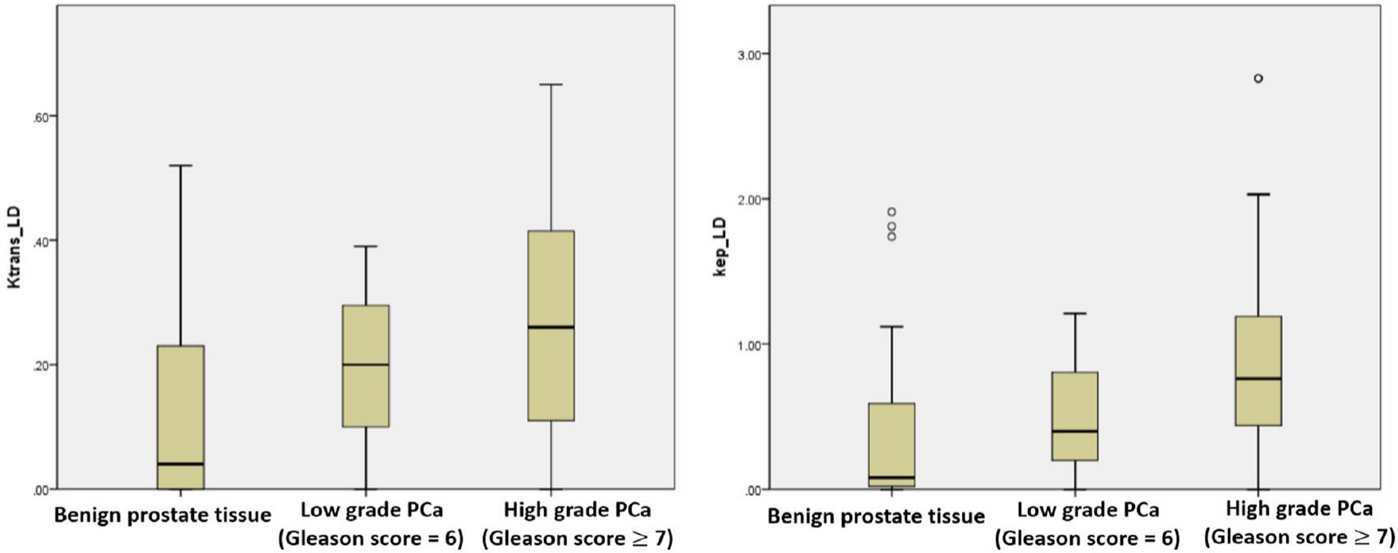
The boxplots of Ktrans _LD and kep_LD showing performance in low grade vs. high grade prostate cancer

Further analysis using a histopathological map of radical prostatectomy specimen slices from 41 LRP patients as a second reference standard confirmed high AUC values of the models (Figure 4). The values of *K^trans^* and *k_ep_* of the LD model showed increasing value, with a corresponding increase in the Gleason score obtained from histological analysis (Figure 5, *p_Ktrans_LD_*=0.02, *p_kep_LD_*=0.01). In addition, the *K^trans^* of the LD model illustrated a relatively high two-tailed bivariate correlation coefficient (0.623) in comparison to the Gleason grade of histopathology obtained using molds fabricated with 3D printing.

## DISCUSSION

Our study has shown a clear distinction of *K^trans^* and *k_ep_* values between benign prostate tissue and prostate cancer (Gleason score≥3+3), similar to several previous studies [10, 15, 16]. Moreover, the *K^trans^* value of the LD model presents good performance accuracy in distinguishing low-grade PCa (Gleason score = 3+3) from high-grade PCa (Gleason score ≥ 3+4) disease (0.272 ± 0.107, 0.323 ± 0.145). This may explain why *K^trans^* has been considered to be the most cogent DCE quantitative parameter in many previous studies [2]. The findings of the present study, with imaging obtained in biopsy naive patients and further confirmation in radical prostatectomy specimens using patient-specific molds for histopathology analysis, adds further knowledge to our understanding. A few previous studies have focused on the semi-quantitative parameters in DCE-MRI [17, 18]. But the analysis of quantitative parameters in DCE-MRI provides the quantitative criterion as a reference rather than relying only on the shape of the DCE-curve, as is used for diagnosis of prostate cancer [15]. Many studies had shown the elevation of *K^trans^*, *k_ep_* and *v_e_* in prostate cancer, as opposed to normal tissue [19–21]. The results of DCE-MRI quantitative parameters from many studies are summarized in Table 2. As can be seen, there is poor agreement regarding the cut-off values. Our results of the mean and cut-off value of *K^trans^* and *k_ep_* are in agreement with some of the previous studies [10, 15, 16, 19–25]. For example, the result of the ET model is similar to that of Ocak et al [21]; the cut-off values show homogeneity with Echo et al [15]. Variations in cut-off values can be explained by many factors. The MRI scanners were produced by different manufacturers, different MRI parameters (time, resolution) during DCE-MRI were used, different patient groups with various stages of prostate cancer were studied, and most importantly, different post-processing software algorithms for DCE-MRI were used. Different software programs provide different models and algorithms for the DCE-MRI data. Even if the DCE-MRI data is processed by the same model, two different software programs can generate different results, as well as *K^trans^* and *k_ep_* maps. The ET model is widely used in many studies [15, 25].

**Table 2:** MRI acquisition parameters

	T1WI	High resolution T2WI			DWI		DCE
	Axial	Sagittal	Axial	Coronal	DWI	DWI high b-value	Dyn Gd-MRI
**Sequence**	2DTSE	2DTSE	2DTSE	2DTSE	2DEPI	2DEPI	3D VIBE
**TR (ms)**	650	6000	4000	5000	3300	3300	4.76
**TE (ms)**	11	102	100	100	95	95	2.45
**Flip angle (°)**	150	140	150	150	-	-	10
**Slice thickness (mm)**	3	3	3	3	3	3	3
**Slice gap (mm)**	0.6	0.6	0.6	0.6	0	0	0.6
**Resolution (pixels)**	320	320	320	320	192	192	192
**FOV (mm)**	200	200	200	200	280	280	280
**b-values (s/mm^2^)**	-	-	-	-	50,100,500,1000	2000	-
**Temporal resolution (s)**	-	-	-	-	-	-	4

Various studies have shown that the combination of T2, DWI and DCE-MRI can improve the detection of prostate cancer [2, 4]. The T2 and DWI scores showed a high specificity of prostate cancer differentiation, which represents the ability to correctly identify healthy people who do not have prostate cancer [26]; however, the sensitivity is relatively low, which means T2 and DWI alone may not be accurate in identifying patients with prostate cancer [27]. The combination of T2 and DWI scores did not improve the sensitivity and specificity. The PI-RADS system also had similar diagnostic accuracy to T2 and DWI. However, with the addition of the DCE cut-off value to PI-RADS as a new grading system, the values of AUC increased by about 10% and the sensitivity was dramatically increased from 56.6% to 92.1%, 93.1%, 91.0% and 89.4% for different models, without a significant decrease in specificity (78.6% to 54.3%, 57.9%, 59.6% and 52.9% for different models). This strongly supports the diagnostic performance of the cut-off value of quantitative DCE parameters, and suggests this grading system has increased diagnostic accuracy compared to the PI-RADS system.

The Gleason score obtained from the second reference standard also validated the diagnostic performance of this grading system. The histological results of whole-mount prostatectomy specimens are considered as the gold standard of PCa diagnosis with higher diagnostic accuracy than biopsy results. With this reference standard, the Gleason score obtained from TRUS-guided biopsy has sensitivity and specificity of 57.3% and 76.9% (p<0.001). This may be due to the fact that TRUS-guided biopsy has poor performance in sampling from anteriorly located tumors, as well as posteriorly located tumors in the peripheral zone [28]. The inhomogeneity of the tumor can also lead to a reduction in the biopsy’s accuracy.

There are several key differences between the present study and the reported literature. Firstly, four different pharmacokinetic models from the same software were used and compared; the cut-off values of the ET and LD models were chosen due to their higher sensitivity and specificity and both of them have shown similarity with some other studies. Secondly, in a subgroup of the cohort, we used our recently published method of providing improved orientation between radiological data and histopathology slides using patient-specific prostate molds [28], which is believed to have a higher diagnostic accuracy. All men in this study had pre-biopsy 3T MRI scanning which avoids any potential artefacts due to biopsies.

In addition to above, present study has limitation. Biopsy accuracy needs to be improved with more state-of-art techniques, e.g. shear wave elastography, MRI fused imaging and multiple-plane imaging transducers. DCE-MRI parameters need to validate with these biopsy techniques. Centre observation needs further external validity including cut-off values described. Research needs to focus areas such as selection and follow-up of men opting for active surveillance using quantitative DCE-MRI parameters as there was clear distinction between findings of high vs. low grade cancers atleast in two models.

## MATERIALS AND METHODS

### Patient recruitment

Between February 2015 and March 2017, 150 patients with suspected localized prostate cancer were recruited into a peer-reviewed protocol-based prospective study (Figure 1). Inclusion criteria, exclusion criteria and baseline characteristics are summarized in Table 3. The prostate volumes were calculated using the formula for a conventional ellipse: maximum diameters of axial × transverse × longitudinal × 0.52 [13]. All participants received a pre-biopsy MRI. The MRI images were reported by an experienced radiologist (MB) and were used to guide DCE-MRI data analysis using MRI post-processing software.

**Table 3:** The mean and SD values of *K^trans^* and *k_e_*_*p*_ in four different kinetics models between benign prostate tissue and PCa (Gleason score≥3+3)

		Tofts & Kermode		Extended Tofts		Lawrence & Lee		Lawrence & Lee Delayed	
		*K^trans^*	*K_ep_*	*K^trans^*	*K_ep_*	*K^trans^*	*K_ep_*	*K^trans^*	*K_ep_*
**Mean, SD (min^−1^)**	**Non-cancer**	0.65±0.52	1.84±1.46	0.27±0.35	0.72±0.66	0.19±0.17	0.6±0.64	0.19±0.17	0.67±0.74
	**cancer**	0.98±0.44	2.57±1.29	0.53±0.44	1.33±0.74	0.29±0.14	0.85±0.44	0.31±0.14	0.99±0.50
**Area under curve**		0.690	0.645	0.736	0.754	0.728	0.674	0.749	0.698
**Cut-off point (min^−1^)**		0.495	1.295	0.205	0.665	0.195	0.47	0.205	0.63
**Sensitivity & Specificity**		91.6%, 48.0%	88.0%, 43.2%	81.7%, 60.1%	88.0%, 62.8%	73.8%, 66.1%	89.0%, 47.8%	78.0%, 67.2%	79.6%, 61.7%
***p* value**		<0.001	<0.001	<0.001	<0.001	<0.001	<0.001	<0.001	<0.001

The cut-off is exported with the sensitivity and specificity.

### MR protocol

All MR images were carried out on 3T scanner (TIM Trio, Siemens, Erlangen, Germany). MR imaging was accomplished 1-2 weeks before 12 core transrectal ultrasound (TRUS) guided biopsies. The mpMRI protocol was derived from the European Society of Uro-radiology Guidelines 2012 for the detection of prostate cancer and subsequent publication of version 2 [13, 14]. Table 4 briefly summarizes the MRI acquisition parameters. Localizer images were acquired in all three imaging planes, where the plane of the prostate was defined in relation to the rectal wall. All subsequent images were acquired in the sagittal, axial or coronal oblique orientations relative to the anatomy of the prostate for each individual (Table 4). All patients were given 1 mL of Hyoscine Butylbromide (Buscopan), 20 mg/mL intravenously prior to MRI, in order to eradicate movement associated with the bowel and rectum in the vicinity of the prostate. The DCE images were obtained using a 3D volume interpolated breath-hold examination (VIBE) gradient echo sequence, with a spine array and 18 channel body-matrix RF coils (Siemens, Erlangen, Germany). The temporal resolution of each volume acquisition was 5.1 seconds and 50 measurements were acquired in total – giving a complete scan time of 4 minutes and 15 seconds. The contrast agent gadoteric acid (Dotarem^©^, Guerbet, Villepinte, France) was administered after the completion of two ‘baseline’ measurements as a volume of 2ml/kg and at a rate of 2ml/second using a Medrad Spectris Solaris EP injector pump (Bayer AG, Leverkusen, Germany). A saline bolus of 20ml was subsequently infused at a rate of 2ml/second following the contrast agent infusion.

**Table 4:** Literature review illustrating heterogeneity in cut-off values [10, 15, 17, 22–28]

	MRI scanner		Post-processing		*K^trans^*(min^−1^)			*K_ep_* (min^−1^)		
	Tesla	Manufacturer	Software	Model	Benign	Cancer	Cut-off	Benign	Cancer	Cut-off
Kozlowski P et al.	1.5T	GE Healthcare	Mat lab	TK	0.60	1.26	-	-	-	-
Ocak l et al.	3T	Philips Medical systems	PRIDE software, Philips	TK	0.23	0.47	-	0.80	1.40	-
Dorston FA et al.	1.5T	Siemens	-	Larsson	0.34	0.59	-	0.89	1.48	-
Li C et al	3T	Philips Medical systems	IDL 6.3	ET	0.09	0.32	-	0.72	1.44	-
Schlemmer et al.	1T	Siemens	VAX alpha 3000/500	Brix	-	-	-	0.98	2.75	-
E Cho et al.	3T	Siemens	Tissue 4D (Siemens)	ET	0.09	0.38	0.184	0.57	1.64	0.695
Padhani AR et al.	1.5T	Siemens	MRIW	ET	0.22	0.79	-	0.26	0.45	-
Langer Dl et al.	1.5T	GE Healthcare	Mat lab	TK	0.298	0.253	-	-	-	-
Peng Y et al.		Philips	Mat lab	ET	-	-	0.257	-	-	-
Fusco R et al.	1.5T	Siemens	Mat lab	ET	-	-	0.14	-	-	-
Our study	3T	Siemens	Olea Sphere	ET	0.64	0.99	0.205	1.83	2.58	0.625
				LD	0.18	0.31	0.205	0.68	0.99	0.63

### Image and histopathology data accrual and analysis

**Figure 6:**
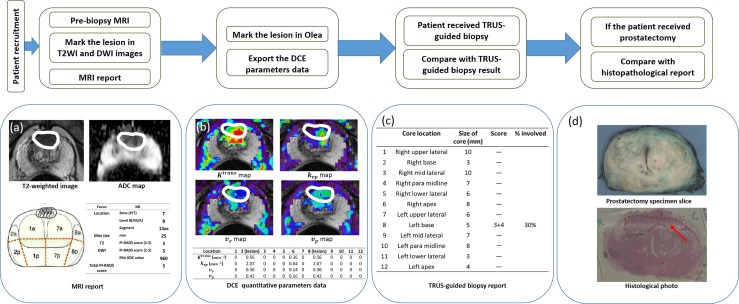
Schematic description of the study, **(a)** T2WI, ADC map and MRI report from MRI examination with marked lesion in black circle, any suspicious lesions had PI-RADS 3 or above were marked. (in 138 patients). **(b)** DCE-MRI quantitative parameters map in Olea. The prostate was divided in 12 regions based on TRUS-guided biopsy cores, any regions contains suspicious lesions in MRI were marked (black circle) in Olea based on the MRI report and the regions without the lesions were marked across the whole area of regions. Then the quantitative DCE data of each region can be obtained. **(c)** The 12-core TRUS-guided biopsy report, the Gleason score obtained from each biopsy region can be compared with the quantitative parameters data (1656 biopsy regions). **(d)** Histopathology photographs were considered as another reference standard of Gleason score for the verification of the comparison between quantitative DCE data and biopsy reports. The Gleason score obtained from each histological region can be compared with the quantitative DCE data as well. 41 patients had the histological reports after received laparoscopic radical prostatectomy. (492 histological regions).

Data acquisition was performed in four stages, namely: (i) initial radiological scoring by experienced uro-radiologists (using T2, DWI and DCE qualitative), (ii) pharmacokinetic modelling assessment from DCE-MRI, (iii) TRUS biopsy analysis and (iv) the histology report of radical prostatectomy in 41 men (Figure 6).

#### Initial MRI scoring

The mpMRI images were analyzed and scored by experienced uro-radiologists using PI-RADS v2.0; all patients’ pathology results were blinded to the radiologist. At least two radiologists read the images (one in multidisciplinary meetings) with good inter-observer agreement. Suspicious lesions which had a PI-RADS score of 3 or above were marked. The size and extent of the lesions identified as suspicious for prostate cancer were recorded.

#### DCE-MRI data analysis

The quantitative parameters were analyzed using different pharmacokinetic models, in accordance with the principle that the contrast agent moves to the extravascular space [10]. The transfer from plasma to extravascular extracellular space (EES), which is the vascular permeability, can be obtained through the movement of the contrast agent [11]. The quantitative parameters include: *K^trans^* (influx transfer constant), *K_ep_* (efflux rate constant), *v_e_* (fractional volume of EES) and *V_p_* (fractional volume of plasma space). The flux moves from the intravascular space to the EES depending on *K^trans^*. The efflux rate constant is the ratios of the influx transfer constant to the fractional volume of EES, which these two parameters are all related to the fundamental physiology [11].

The DCE-MRI data were analyzed using advanced MRI post-processing software, Olea Sphere (Olea Medical, La Ciotat, France). Based on the MRI report, the regions of interest (ROI) were marked on the DCE-MR images in Olea Sphere v2.3. For all the regions with suspicious lesions, ROIs were drawn around the lesion. In the regions without suspicious lesions, ROIs were drawn around the whole area. The MRI time-signal enhancement curves in each voxel were fitted in accordance with a pharmacokinetic model. In Olea Sphere, motion correction was performed automatically at first. Following that, four different models (Tofts & Kermode, extended Tofts, Lawrence & Lee, Lawrence & Lee Delayed) could be selected; the first two models (TK, ET) were widely used in clinical areas, while the other two models (LL, LD) have been used for research. After the model was selected, the color map of various parameters was automatically computed. Hence, for each region, and four quantitative parameters (*K^trans^*, *k_ep_*, *v_e_*, *v_p_*) of each modelwere calculated and obtained.

The prostate MRI images were divided into 12 regions to correlate with the standard 12-core TRUS guided biopsy (right upper lateral, right base, right mid lateral, right para midline, right low lateral, right apex, left upper lateral, left base, left mid lateral, left para midline, left lower lateral, left apex). All regions with or without suspicious lesions inside the areas were analyzed and compared with the 12-core TRUS guided biopsy results for the whole cohort and in a sub-cohort histopathology of radical prostatectomy specimen.

#### TRUS biopsy data

The biopsy results were analyzed by experienced pathologists; who were each blinded to radiology results. The location and size of the core, the Gleason score and the degree of core involvement were acquired. Given this information, the quantitative DCE-MRI results of each region could be compared to the biopsy results.

#### Histopathology of radical prostatectomy specimens (validation cohort)

This was carried out only on a subset of the cohort. 41 patients who underwent laparoscopic radical prostatectomy (LRP); the radical prostate specimens for histology were sliced in patient-specific molds, which were fabricated using a 3D printer. In the process of sectioning, a single blade was applied carefully to prevent the friction and shifting of the specimen. Then all the sections were photographed and analyzed by pathologists. The technique has been described by us previously [28]. The histopathology map of each section was then compared with the MRI images and post-processing analysis. The histopathology maps were divided into 12 regions as described in an earlier approach for TRUS biopsies. The Gleason score obtained from the histopathology were used as a second reference standard for the validation of the results from the biopsy findings.

### Statistical analysis

All analyses were performed using the Statistical Package for Social Science (SPSS) software (version 22). The values of quantitative DCE-MRI parameters were compared with histopathology. The differences between mean and standard deviation (SD) values of quantitative parameters in normal and prostate cancer were calculated. Receiver-operator-characteristic (ROC) curves were performed for the evaluation of diagnostic performance of the DCE parameters. Area under curve (AUC), sensitivity and specificity of each ROC curve were also calculated. Based on these values, the Youden index could be obtained. The point with the highest Youden index was extracted from the ROC curves as the cut-off point, which was determined to be the most accurate value for prostate cancer differentiation. The differences of quantitative parameters between low-grade PCa (Gleason score=3+3) and high-grade PCa (Gleason score ≥ 3+4) were calculated as well. A p-value of <0.05 was considered significant.

## CONCLUSIONS

In conclusion, quantitative DCE-MRI parameters improve the diagnostic performance of MRI in distinguishing normal prostate tissue from prostate cancer and its various grades. The cut-off values of *K^trans^* and *k_ep_* of the ET and LD models were defined, which could act as potential markers of the disease.
